# Exclusive breastfeeding practice in Ethiopia and its association with antenatal care and institutional delivery: a systematic review and meta-analysis

**DOI:** 10.1186/s13006-018-0173-x

**Published:** 2018-07-16

**Authors:** Animut Alebel, Cheru Tesma, Belisty Temesgen, Aster Ferede, Getiye Dejenu Kibret

**Affiliations:** 1 Department of Nursing, College of Health Sciences, Debre Markos University, Debre Markos, Ethiopia; 2 Department of Public Health, College of Health Sciences, Debre Markos University, Debre Markos, Ethiopia; 3 Debre Markos Referral Hospital, Debre Markos, Ethiopia

**Keywords:** Prevalence, Exclusive breastfeeding, Meta-analysis, Systematic review, Ethiopia

## Abstract

**Background:**

Despite the World Health Organization recommendation of exclusive breastfeeding (EBF) for the first six months of life, the rate remains low both in developed and developing countries. In Ethiopia, findings regarding the prevalence of EBF have been highly variable. Antenatal care and institutional delivery are the most important factors contributing to the practice of EBF however; their effect has not been investigated in Ethiopia.

**Methods:**

In this systematic review and meta-analysis, international databases were systematically searched. All observational studies reporting the prevalence of EBF and its association with antenatal care and institutional delivery in Ethiopia were considered. Two authors independently extracted all necessary data using a standardized data extraction format. A random effects meta-analysis model was computed to estimate the pooled prevalence of exclusive breastfeeding. Moreover, the association of antenatal care and institutional delivery with EBF was determined.

**Results:**

After reviewing 619 studies, 32 studies fulfilled the inclusion criteria and were included in the meta-analysis. The pooled prevalence of EBF in Ethiopia was 59.3% (95% Confidence Interval [CI] 53.8, 64.8). The subgroup analysis indicated that the highest prevalence was observed in Afar region (65.6%), followed by SNNP (63.8%), and then by Oromia (61.8%). Additionally, mothers who attended antenatal visits were 2.1 times more likely to practice EBF compared to their counterparts (Odds Ratio [OR] 2.1; 95% CI 1.5, 2.8). Moreover, mothers who gave birth at a health institution were 2.2 times more likely to practice EBF compared to mothers who gave birth at home (OR 2.2; 95% CI 1.3, 3.5).

**Conclusions:**

Exclusive breastfeeding in Ethiopia was significantly lower than the global recommendations. There was evidence that mothers who attended antenatal visits and who gave birth at health institutions had better EBF practices. Based on our findings, we strongly recommended that the utilization of antenatal care and institutional delivery should be improved through health extension workers.

## Background

Breast milk is the natural first food for babies, which provides all the energy and nutrients that the infant needs for the first 6 months of life [1]. The World Health Organization (WHO) recommends that infants should be exclusively breastfed for the first 6 months, and for an additional 18 months or longer, to be breastfed along with complementary foods for the achievement of satisfactory growth and development [2, 3]. Exclusive breastfeeding (EBF) is defined as giving breast milk to the infant, without any additional food or drink, not even water in the first 6 months of life, with the exception of vitamins, mineral supplements or medicines [4].

Although the benefit of exclusive breastfeeding is widely advocated globally, only 35% the of infants worldwide were exclusively breastfed during their first 4 months of life [5, 6]. In Africa, Asia, Latin America, and Caribbean countries, evidences suggested that only 47–57% of infants less than 2 months, and 25–31% of infants 2–5 months were exclusively breastfed [7]. Even though, Sub-Saharan Africa has one of the highest prevalence of breastfeeding at 1 year worldwide; however, only 37% of infants aged less than 6 months are exclusively breastfed [8]. Non-exclusive breastfeeding has been significantly associated with increased infants and young child mortality. Accordingly, almost 96% of all infant deaths that means 1.24 million deaths occur during the first 6 months of life are attributed to non-exclusive breastfeeding, this figure is substantially higher in Asian and African countries. In addition, non-exclusive breastfeeding also contributes to 55% of diarrheal deaths and 53% of acute respiratory deaths for the first 6 months of life [9]. Partial or no breastfeeding is associated with a 2.23-fold higher risk of infant deaths resulting from all causes and 2.40 and 3.94 fold higher risk of deaths attributable to pneumonia and diarrhea, respectively as compared with exclusive breastfeeding [10]. In Ethiopia, suboptimal breastfeeding contributes to an estimated number of 70,000 infant deaths, which accounts 24% total infant death annually. These deaths could be prevented through nutritional interventions like exclusive breastfeeding [11].

Antenatal Care (ANC) is one of the fundamental strategies recommended to reduce the risk of maternal and neonatal mortality both in developing and developed countries [12–15]. It is an ideal entry point for healthcare professionals to provide numerous healthcare interventions to promote maternal and fetal wellbeing including exclusive breastfeeding [16]. During ANC visits, different nutritional and other health related educations from healthcare professionals are provided, which could have a great contribution to the practice of exclusive breastfeeding. In addition, information provided by healthcare professions concerning infant feeding and the nutritional values of breast milk will increase the knowledge of exclusive breastfeeding [16]. According to WHO, to provide an effective ANC service of at least four ANC visits are recommended particular to low-income countries [17]. Moreover, an institutional delivery encourages infants to receive skin to skin contact from their mothers, and this will increase the likelihood of timely initiation of breastfeeding, exclusive breastfeeding and prolonged duration of breastfeeding [18, 19]. Furthermore, institutional delivery has a great role to promote breastfeeding through a strategy of the Baby-Friendly Hospital Initiative (BFHI). The BFHI is a key component of the World Health Organization/United Nations Children’s Fund Global Strategy for Infant and Young Child Feeding [20].

In Ethiopia, different studies have been conducted to determine the prevalence of EBF and associated factors. The findings of these fragmented studies documented that there was a great variability in the prevalence of EBF across the regions of the country. Regarding associated factors, these studies revealed that different maternal and health service related factors influenced EBF; maternal educational level [21–26], current marital status [27, 28], place of residence [21, 29, 30], employment [21, 25, 28, 30–37], economic status of mothers [30, 31, 37, 38], institutional delivery [21, 23, 25, 27, 37, 39–42], history of antenatal [22, 29, 33, 37, 38, 43–45] and postnatal care [22, 23, 25, 29, 33, 34, 37, 39, 43], were some of the factors associated with EBF practice. From these factors, we selected the two factors (ANC and institutional delivery) to see their effect on the practice of exclusive breastfeeding. We sorted these factors because of the following reasons: firstly, these factors were the most important factors, which ultimately influencing EBF. Secondly, the effect of ANC and institutional delivery on EBF have reported controversial findings. Regarding these findings, in some studies, ANC was positively associated with EBF [22, 24, 28, 29, 31, 37, 39, 40, 42, 45–48]. On the other hand, one Ethiopian study disclosed that ANC was negatively associated with EBF [49]. Regarding institutional delivery, in some studies it was positively associated with EBF [21, 25, 28, 30, 31, 36, 37, 39, 40, 42, 43, 45, 47, 50, 51]. On the other hand, in two studies it was inversely associated with EBF [41, 44]. Therefore, the above-mentioned factors required this meta-analysis.

For better intervention, current and up-to-dated information regarding the prevalence of EBF and its association with ANC and institutional delivery is vital, especially in low and middle-income countries like Ethiopia. However, despite these small and fragmented studies there was no nationwide study, which determines the prevalence of EBF and its association with ANC and institutional delivery in Ethiopia. Hence, the main aim of this systematic review and meta-analysis was to estimate the pooled prevalence of EBF and its association with ANC and institutional delivery in the context of Ethiopia. The findings of this study will be an input to policy makers and program planners working in the area of breastfeeding.

## Methods

### Searching strategies

The current systematic review and meta-analysis was reported by using the Preferred Reporting Items for Systematic Reviews and Meta-Analysis (PRISMA) [52] guideline to determine the pooled prevalence of exclusive breastfeeding practice and its association with ANC and institutional delivery in the context of Ethiopia. The international databases, including PubMed, Google scholar, Science direct and Cochrane library were systematically searched. The search was conducted using the following keywords “prevalence”, “Exclusive breastfeeding”, “Antenatal care”, “Institutional delivery”, “Place of delivery”, “Birth place”, “Skill birth attendance”, and “Ethiopia”. The search terms were used separately and in combination using Boolean operators like “OR” or “AND”. The search was conducted from September 9 to October 10, 2017. All papers published until October 10, 2017 were included in this review.

### Eligibility criteria

#### Inclusion criteria

Study area: Only studies conducted in Ethiopia.

Publication condition: Articles published in peer reviewed journals.

Study design: All observational study designs (Cross-sectional, case-control and cohort) reporting the prevalence of EBF or studies reporting the associations between ANC and institutional delivery with EBF were considered.

Outcome of interests: Studies reported data on the prevalence of EBF or the association between EBF and ANC or the association between EBF and institutional delivery were considered.

Language: Articles reported in the English language were considered.

#### Exclusion criteria

Articles, which were not fully accessed, after at least two email contact of the primary author were excluded. Exclusion of these articles is because of inability to assess the quality of articles in the absence of full text.

### Outcome measurement

This systematic review has three main outcomes. Exclusive breastfeeding practice, as the primary outcome variable of this study, is defined as giving breast milk to the infant, without any additional food or drink, not even water in the first 6 months of life, with the exception of vitamins, mineral supplements or medicines [4]. The prevalence was calculated from each primary study by dividing the number of women breastfeeding exclusively to the total number of women who had ever breastfed multiplied by 100. The second outcome was to examine the association between ANC and exclusive breastfeeding. In this study, healthcare professionals considered antenatal care as if a mother received at least four antenatal care visits and more during her pregnancy period. The third outcome of this study was to determine the association between institutional delivery and exclusive breastfeeding. Institutional delivery was defined as giving birth at the health facility. For the second and the third outcomes, we calculated the log odds ratio based on the primary studies that examined the relationship between ANC, institutional delivery with exclusive breastfeeding.

### Data extraction

Data were extracted using a standardized data extraction format, which was adopted from the JBI data extraction format. Two authors (AA and CT) independently extracted all necessary data using the format. Any disagreements at the time of data abstraction were resolved through discussion and consensus**.** The data extraction format included primary author, publication year, study area, study design, response rate, sample size, prevalence with 95% CI and the quality score of each study.

### Quality assessment

The Newcastle-Ottawa Scale for cross-sectional studies quality assessment tool was adapted and used to assess the quality of each study [53]. The tool has three major sections. The first section graded from five stars focuses on the methodological quality of each study. The second section of the tool deals with the comparability of the study. The last section deals with the outcomes and statistical analysis of each original study. Two authors independently assessed the quality of each original study using the tool. Disagreements between the two authors were resolved by taking the mean score of the two authors. Finally, research with a scale of ≥6 out of 10 were considered as high quality.

### Statistical analysis

Data were extracted in Microsoft Excel format, then analysis was done using STATA version 13 statistical software. The standard error for each original study was calculated using the binomial distribution formula. Heterogeneity among reported prevalence was assessed by computing *p*-values for Cochrane Q-statistics and *I*^2^ test [54]. As the test statistic showed there was a significant heterogeneity among the included studies (*I*^2^ = 98.7%, *p* < 0.001) as a result a random effects meta-analysis model was used to estimate the Der Simonian and Laird’s pooled effect. To minimize the random variations between the point estimates of the primary study subgroup, analysis was done based on study settings (i.e., the area where studies were conducted) and sample size. In addition, to identify the possible sources of heterogeneity univariate meta-regression was conducted by considering the sample size and year of publication as covariates but none of them were found to be statistically significant. Furthermore, Egger’s and Begg’s tests at 5% significant level were not significant for publication bias [55]. Point prevalence as well as 95% confidence intervals were presented in the forest plot format. In this plot, the size of each box indicated the weight of the study, while each crossed line refers to 95% confidence interval. For the second and third outcomes, log odds ratios were used to examine the association between ANC and institutional delivery with EBF.

## Results

**Fig. 1:**
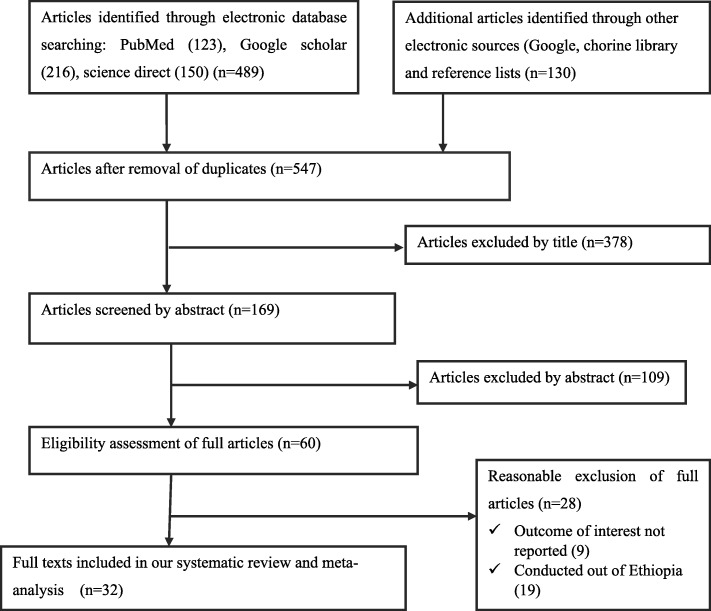
Flow chart to describe the selection of studies for a systematic review and meta-analysis of the effects of ANC and institutional delivery on exclusive breastfeeding in Ethiopia, 2017

As shown in Fig. 1, in the first step of our search, 619 studies were identified regarding EBF in Ethiopia through PubMed, Google Scholar, Science Direct and others. Of which, 72 studies were excluded due to duplicates. From the remaining 547 studies, 378 articles were excluded after reviewing of their titles based on assessment as non-relevance to this review. The remaining 169 studies were screened by abstracts yielding an additional 109 being excluded. Moreover, 60 full text articles were accessed and assessed for eligibility based on the preset inclusion criteria. Of these, 28 articles were excluded due to the inclusion criteria. Among these, nine of the studies were excluded because they didn’t report our outcome of interests [49, 56–62]. The remaining 19 articles were excluded due to study settings; four of the studies were from Ghana [63–66], three of the studies were from Tanzania [67–69], one from Bangladesh [70], two from Cameroon [71, 72], two from India [73, 74], three from Kenya [75–77] one from Nigeria [78], two from Malaysia [79, 80], two from Congo [81, 82], and one from Nepal [83]. Finally, 32 studies fulfilled the inclusion criteria and included in the systematic review and meta-analysis.

### Description of the included studies

As shown in Table 1, these 32 studies were published between 2007 to 2017. In the current meta-analysis, 23,543 breastfeeding women were included to estimate the pooled prevalence of exclusive breastfeeding. Regarding study design, most 29 (71.8%) of the studies are cross-sectional study design. The sample size of the studies ranging from 119 to 5, 227. The lowest prevalence (29.3%) of EBF was observed in a study conducted in Addis Ababa, Ethiopia [43] whereas the highest prevalence (86.1%) was observed in a study conducted in north Gondar zone, northwest Ethiopia [51]. In this meta-analysis, from nine regions of the country seven regions were represented. Thirteen of the studies were from Amhara [23, 25, 29–31, 33, 37, 40, 42, 45, 47, 48, 51], three from Addis Ababa [43, 59, 84], three from Afar [21, 34, 50], four from Oromia [32, 36, 38, 41], six from SNNP [22, 24, 26, 28, 44, 46], two from Tigray [35, 39]. However, there were no studies reported from Benishangul Gumiz and Gagmbela regions. Concerning the quality score, the score ranged from lowest 3 to highest 8. In related to response rate, almost all studies had a good response rate. The possible reason for this high response rate could be due to most of the studies used interviewers administered questionnaire to collect the data (Table 1).

**Table 1:** Descriptive summary of 32 studies included in the meta-analysis of the prevalence of exclusive breastfeeding in Ethiopia, 2017

Primary author	Publication year	Study area	Study design	Response rate (%)	Sample size	Prevalence with 95% CI	Quality score
Adugna et al. [28]	2017	Gozamen district	Cross-sectional	97.8	541	60.9 (56.7, 65.0)	8
Alemayehu et al. [27]	2009	EDHS 2005 based	EDHS 2005 based	NR	1142	49 (46.1, 51.9)	6
Alemu Earsido et al. [26]	2017	Hossana town	Cross sectional	98	720	73.8 (70.6, 77.1)	5
Arage and Gedamu [42]	2016	Debre Tabor	Cross-sectional	96.4	470	70.9 (66.7, 75.1)	6
Asemahagn [37]	2016	Azezo district	Cross-sectional	96	346	78.9 (74.5, 83.3)	8
Asfaw et al. [30]	2015	Debre Berhan District	Cross-sectional	100	634	68.6 (65.0, 72.2)	7
Bekere et al. [36]	2014	West Oromia	Cross-sectional	99.2	119	72 (63.9, 80.1)	5
Berhe et al. [35]	2013	Mekelle town	Cross-sectional	100	361	60.7 (55.6, 65.7)	4
Biks et al. [45]	2015	Dabat district	a nested case-control study	NR	1769	30.7 (28.6, 32.8)	8
Chekol et al. [47]	2017	Gondar town	Cross-sectional	NR	649	34.8 (31.2, 38.5)	8
Genetu et al. [51]	2017	north Gondar zone	Cross-sectional	NR	367	86.1 (82.6, 89.6)	8
Gizaw et al. [50]	2017	Hadaleala district	Cross-sectional	98.5	294	60.6 (54.6, 66.6)	7
Hunegnaw et al. [25]	2017	Gozamin district	Cross-sectional	94.4	506	74.1 (70.1, 78.0)	8
Kitesa and Bekele [41]	2017	Marti district	Cross-sectional	100	2222	44.3 (42.3, 46.4)	6
Lenja et al. [24]	2016	Offa district	Cross-sectional	98	403	78 (74.0, 82.1)	8
Liben et al. [34]	2016	Dubti Town	Cross-sectional	96.2	346	81.1 (76.9, 85.3)	7
Mekuria and Edris [33]	2015	Debre Markos	Cross-sectional	97.6	423	60.8 (56.1, 65.5)	7
Mukerem and Haidar [59]	2012	Addis Ababa	Cross-sectional	966	384	73 (68.5, 77.6)	6
Reddy and Abuka [44]	2016	Dilla Zuria District	Cross-sectional	98.8	352	57.6 (52.4, 62.8)	3
Sefene et al. [23]	2013	Bahir Dar Town	Cross-sectional	93.5	170	49.1 (41.3, 56.8)	5
Seid et al. [40]	2013	Bahir Dar	Cross-sectional	100	819	50.6 (47.1, 54)	7
Seifu et al. [38]	2014	Jimma Town	Cross-sectional	97.2	422	60.2 (55.5, 65.0)	7
Setegn et al. [32]	2012	Goba district	Cross-sectional	91	668	71.4 (67.8, 75.0)	8
Shewayenesh [84]	2007	Yeka sub-city	Cross-sectional	100	796	34 (30.8, 37.3)	4
Shifraw et al. [43]	2015	Addis Ababa	Cross-sectional	98	660	29.3 (25.8, 32.8)	7
Sonko and Worku [22]	2015	Halaba special woreda	Cross-sectional	99.5	422	70.5 (66.1, 74.8)	8
Sorato [46]	2017	Chencha district	Cross-sectional	92	248	40.7 (54.5, 66.4)	6
Tariku et al. [48]	2017	Dabat District	Demographic Surveillance	NR	5227	54.5 (53.2, 55.90	8
Teka et al. [39]	2015	Enderta woreda	Cross-sectional	98	541	70.2 (66.3, 74.1)	7
Tewabe et al. [31]	2017	Motta town	Cross-sectional	95.7	423	50.1 (45.3, 55.0)	7
Tsegaye [21]	2015	Aysaita wereda	Cross-sectional	98	631	55 (51.1, 58.9)	6
Woldie et al. [29]	2014	Mecha district	Cross-sectional	100	819	47.1 (43.7, 50.6)	7

### Meta–analysis

**Fig. 2:**
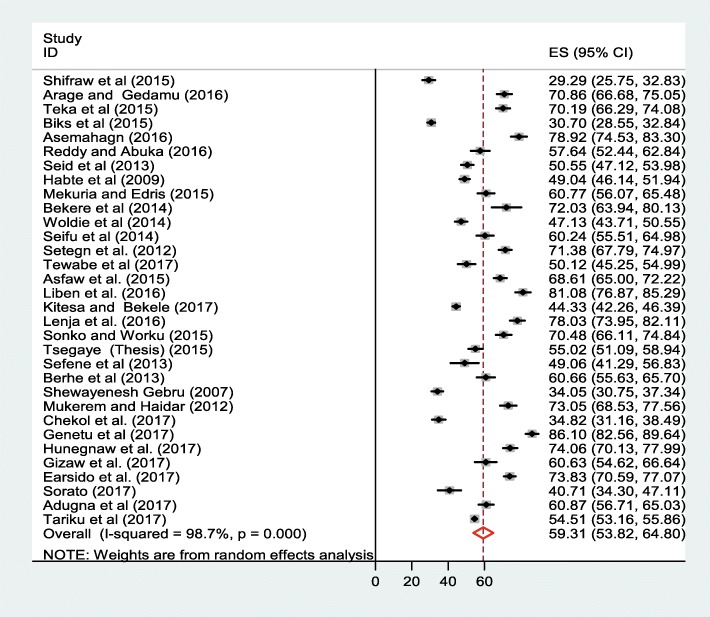
Forest plot of the pooled prevalence of exclusive breastfeeding in Ethiopia

The result of 32 included studies indicated that the pooled prevalence of EBF in Ethiopia was 59.3% (95% CI: 53.8, 64.8%) (Fig. 2). In this meta-analysis, Genetu et al. (86.1%) reported the highest prevalence of EBF, whereas Shifraw et al. (29.3%) reported the lowest prevalence of exclusive breastfeeding. The result of univariate meta-regression models was presented in the table below (Table 2). Publication bias was assessed using Begg’s and Egger’s tests, showing no statistical significant for estimating the prevalence of EBF (*p* = 0.5) and (*p* = 0.2) respectively.

**Table 2:** Related factors with heterogeneity of exclusive breastfeeding prevalence in the current meta-analysis (based on univariate meta-regression)

Variables	Coefficient	*p* - value
Publication year	1.01	0.76
Sample size	0.0007	0.84

### Subgroup analysis

In addition, in this meta-analysis, we performed subgroup analysis based on the regions where the studies were conducted and sample size of the studies. Accordingly, the highest prevalence was reported in Afar region with a prevalence of 65.6% (95% CI: 48.5, 82.7%) followed by SNNP, 63.8% (95% CI: 54.6, 73.0%) and Oromia, 61.8% (95% CI: 46.1, 77.6%). With regard to sample size, the prevalence of EBF was higher in studies having a sample size < 600, 66.2% (95% CI: 61.2, 71.3%) compared to those having a sample size ≥ 600, 49.0% (95% CI: 40.5, 57.6%) (Table 3).

**Table 3:** The subgroup prevalence of exclusive breastfeeding in Ethiopia, 2017 (*n* = 32)

Variables	Characteristics	Included studies	Sample size	Prevalence (95% CI)
By regions	Addis Ababa	3	1802	45.4 (21.1, 69.7)
	Amhara	13	12, 520	58.2 (49.0, 67.3)
	Oromia	4	3358	61.8 (46.1, 77.6)
	Afar	3	1, 205	65.6 (48.5, 82.7)
	SNNP	6	2, 625	63.8 (54.6, 73.0)
	Others	3	2033	59.9 (46.3, 73.5)
By sample size	≥ 600	13	16,641	49.5 (42.1, 56.8)
	< 600	19	6, 902	66.2 (61.2, 71.3)
Overall		32	23,543	59.3(53.8, 64.8)

### The linear trend of exclusive breastfeeding in Ethiopia

**Fig. 3:**
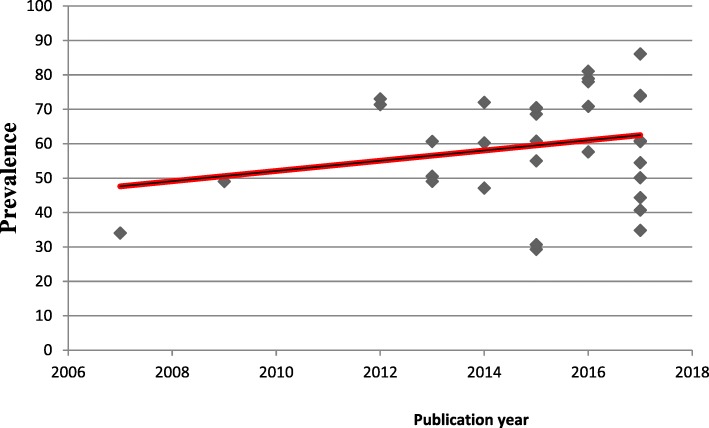
The linear trend of exclusive breastfeeding in Ethiopia from 2007 to 2017

In this study, we also describe the linear trend of exclusive breastfeeding in Ethiopia from 2007 to 2017. We found that the general linear trend of exclusive breastfeeding was increased in each successive year (Fig. 3).

### Association between ANC and exclusive breastfeeding

**Fig. 4:**
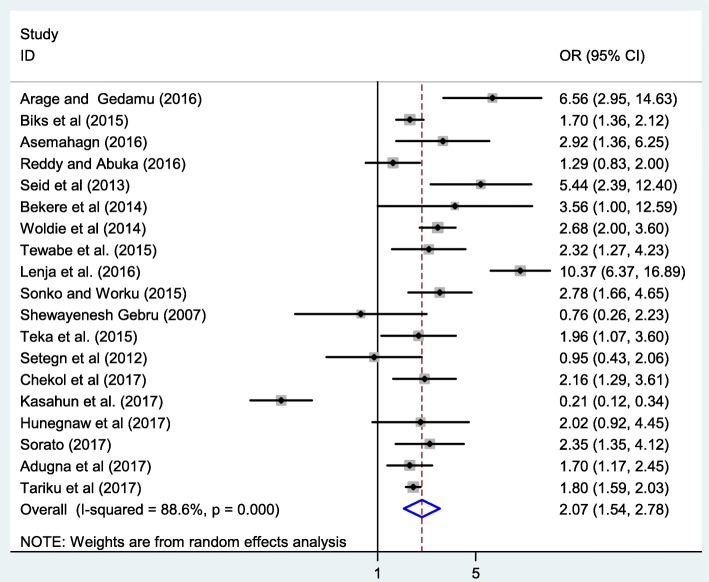
The pooled odds ratio of the association between ANC and exclusive breastfeeding in Ethiopia

In this meta-analysis, we examined the association between ANC and EBF practice by using nineteen available studies [22, 24, 25, 28, 29, 31, 32, 36, 37, 39, 40, 42, 44–49, 84]. The findings from these nineteen studies revealed that the practice of EBF was significantly associated with antenatal care. Accordingly, the likelihood of EBF practice was 2.1 times higher among mothers’ who had ANC visits as compared to their counterparts (OR: 2.1, 95 %CI: 1.5, 2.8) (Fig. 4). High heterogeneity (*I*^*2*^ = 88.6% and *p* - value < 0.001) was observed across the included studies; hence, a random effect meta-analysis model was used to examine the association between ANC and EBF. For this analysis, we also assessed publication bias using Begg’s and Egger’s tests, the result of the test statistics indicated that there was no possible presence of statistically significant publication bias (*p* = 0.4 and (*p* = 0.6) respectively.

### Association between institutional delivery and exclusive breastfeeding

**Fig. 5:**
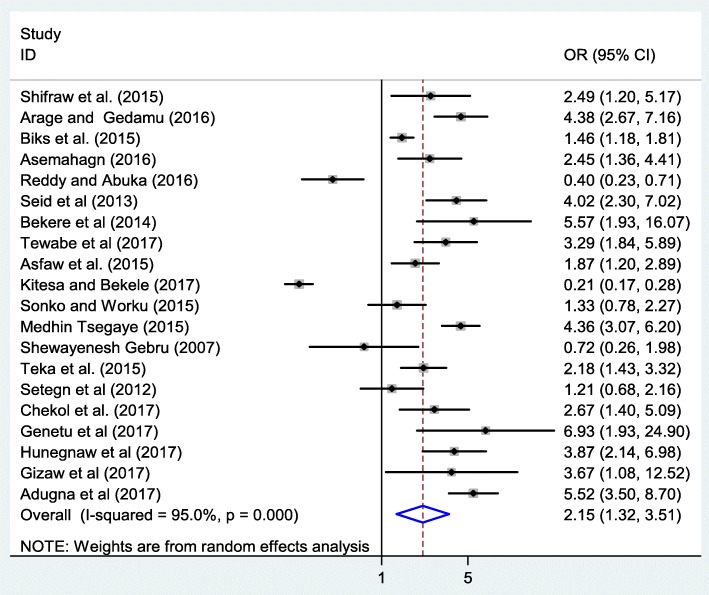
The pooled odds ratio of the association between institutional delivery and exclusive breastfeeding in Ethiopia

The third outcome of this research was to determine the association between institutional delivery and exclusive breastfeeding. Twenty studies, which examined the association between institutional delivery and EBF were considered to determine the association between EBF practice and institutional delivery [21, 22, 25, 28, 30–32, 36, 37, 39–45, 47, 50, 51, 84]. In this study, the pooled odds ratio indicated that institutional delivery was positively associated with EBF (OR: 2.2, 95% CI: 1.3, 3.5) (Fig. 5). In this meta-analysis, extreme heterogeneity (*I*^2^ = 95.0% and *p* - value < 0.001) was observed across the studies hence, a random effect meta-analysis model was employed to estimate the pooled effect. Moreover, to detect the presence of publication bias, we did Begg’s and Egger’s tests. However, none of the tests were revealed significant publication bias with *p* - value of 0.8 and 0.08 respectively.

## Discussion

We conducted this systematic review and meta-analysis to estimate the pooled prevalence of EBF practice in Ethiopia and its association with ANC and institutional delivery. In Ethiopia, conducting this type of study will be paramount as input for program planners and policy makers working in the area of breastfeeding. The result of 32 included studies noted that the pooled prevalence of EBF in Ethiopia was 59.3% (95% CI: 53.8, 64.8%). The overall prevalence indicated in this meta-analysis is higher than a meta-analysis conducted in Iran (49.1%) [85]. In addition, this finding is higher than the national prevalence reported from other Sub-Saharan African countries included: Cameroon (28.2%), Nigeria (17.4%), Benin (43.1%), Burkina Faso (50.1%), and Ghana (52%), [86]. On the other hand, our finding is slightly lower than the national prevalence of Malawi (61.2%), Kenya (61.4%), and Peru (68.4%) [86]. The possible explanations for the above variations could be due to methodological differences (i.e., data analysis and sampling of study participants), variation in infants and maternal sociodemographic characteristics, economical, and health service utilization. Moreover, the higher prevalence of EBF in this meta-analysis could be attributed to the implementation of different strategies by the Ethiopian Government including the Health Extension Program [87].

In this study, we also performed sub-group analysis based on the study areas (i.e. Regions of the country) where the studies were conducted. The findings of the subgroup analysis indicated that extreme variability was observed in the prevalence of EBF across the regions of the country. The highest (65.6%) prevalence of EBF was reported from the Afar region, whereas the lowest (45.4%) prevalence of EBF was reported from Addis Ababa. The possible explanation for this variation could be due to the cultural variation across the regions of the country. In studies conducted in the Afar region, pastoralist communities were involved [88]. Therefore, the higher prevalence of EBF in Afar region could be due to the norm and culture to breastfeed the babies in the pastoralist communities [21]. Another possible explanation might be the difference in the implementation of health extension program [50]. Moreover, the high prevalence of EBF in Afar region could be also attributed to most of the women spent their time at home. Evidences suggested that mothers working at home is a major enhancing factors of exclusive breastfeeding [89, 90].

In this meta-analysis, we tried to describe the linear trend of exclusive breastfeeding in Ethiopia for the past ten years. From the result, we observed that the practice of exclusive breastfeeding practice was slightly increased from 34% in 2007 to 86.1% in 2017. The possible justification for the increased in the practice of EBF could be due to the fact that, the Ethiopian government has developed and implemented different infants and young child feeding guidelines and giving appropriate emphasis to key messages on exclusive breastfeeding practice since 2004 [91]. Additionally, different interventions are also launched as breastfeeding promotions have been given at health institutions and at the community level by community health extension workers and other healthcare providers [50]. Health information dissemination using different means, the preparation of training manuals and guidelines are some of the intervention strategies launched to enhance exclusive breastfeeding [28]. These factors could have a great contribution for the successive increment of exclusive breastfeeding through each year.

The current meta-analysis was also examined the association between ANC and EBF in Ethiopia context. Accordingly, antenatal care was significantly associated with exclusive breastfeeding. Mothers who had ANC visits were almost 2.1 times more likely to practice exclusive breastfeeding as compared to mothers who hadn’t received antenatal care. This finding is consistent with the studies conducted in Rawalpindi [92] and Singapore [93]. This could be due to the fact that mothers who had ANC visit may receive different nutritional and other health related educations from health professionals during their follow up time these might have a great impact on exclusive breastfeeding [16]. Another conceivable explanation could be the increased knowledge and attitudinal changes due to the information provided by the healthcare professional about infants feeding and the nutritional values of breast milk.

Mothers who gave birth at a health institution were almost 2.2 times more likely to practice exclusive breastfeeding as compared to those who gave birth at home. This finding is in agreement with studies conducted in Ghana [94], India [73], Tanzania [95]. This might be because mothers who gave birth at health institution have a good opportunity to receive postnatal counseling regarding the importance of EBF, good position and attachment of breastfeeding from healthcare professionals. Supportive findings indicated that postnatal counseling regarding EBF have a great contribution to practice exclusive breastfeeding [23]. On the other hand, our finding contradicts with a study reported from Canada [96]. This study indicated that mothers who gave birth at home were more likely to practice EBF as compared to their counterparts. This controversy finding could be explained by socioeconomic and cultural variation across the study participants.

### Limitations of the study

Like other meta-analysis, this meta-analysis has several limitations. The first limitation of this study was only English articles or reports were considered to conduct this nationally based review. In addition, the majority of the studies included in this review were cross-sectional in nature as a result; the outcome variable might be affected by other confounding variables. Moreover, most of the studies included in this review had a small sample size. Therefore, this factor could affect the estimated reports. Furthermore, this meta-analysis represented only studies reported from seven regions of the country. Therefore, the regions may be under-represented due to the limited number of studies included.

## Conclusions

The overall prevalence of EBF practice in Ethiopia was significantly low as compared to the global recommendation level of breastfeeding. Mothers who received ANC and an institutional delivery were significantly associated with the better EBF practice. Therefore, based on our findings, we strongly recommended that healthcare workers (midwives and obstetricians) should give a special emphasis to encourage mothers to attend antenatal and postnatal care to improve EBF practice as an opportunity to teach mothers about the importance of exclusive breastfeeding. In addition, improving utilization of antenatal care and institutional delivery through health extension workers are essential interventions to increase exclusive breastfeeding practice. Moreover, besides the institutional level, health extension workers shall give infant and young children feeding advice/counseling at the community.

## Abbreviations

ANC: Antenatal Care
DHS: Demographic and Health Survey
EBF: Exclusive Breastfeeding
EDHS: Ethiopian Demographic and Health Survey
HSDP: Health Sector Development Plan
OR: Odds Ratio
WHO: World Health Organization
